# A41 THE ROLE OF PGC-1α IN MURINE BONE MARROW DENDRITIC CELLS

**DOI:** 10.1093/jcag/gwab049.040

**Published:** 2022-02-21

**Authors:** S Jaleel, F Lopes

**Affiliations:** McGill University Faculty of Agriculture and Environment, Sainte Anne de Bellevue, QC, Canada

## Abstract

**Background:**

The intestinal immune system tolerates food antigens and the commensal microbiota to prevent chronic inflammation. Inflammatory bowel diseases (IBD) such as ulcerative colitis and Crohn’s disease, are characterized by the loss of tolerance towards the microbiota, leading to inflammation, which is characterized by the high production of cytokines such as TNF and the influx of macrophages and neutrophils to the gut. Tolerance is driven by tolerogenic dendritic cells (tolDCs). Currently, there is no cure for IBD and while available treatments may reduce symptoms, there are no pharmacological therapies that specifically seek to rescue tolerance. Inducing tolDCs in patients with IBD may be a promising therapy since it is a way to directly target the main cause of the disease. tolDCs have been defined by down-regulating TNF (pro-inflammatory cytokine), producing IL-10 (anti-inflammatory cytokine), and active oxidative phosphorylation (OXPHOS) in the mitochondria.

**Aims:**

Given the tolDC characteristic of active OXPHOS, the objective of our project was to evaluate the role of PGC-1α, a major mediator of mitochondria biogenesis, in generating toDCs using murine bone marrow-derived dendritic cells (BMDC). Our hypothesis is that tolDCs would be (1) generated by pharmacological activation of PGC-1α by ZLN005, and (2) constrained by inhibition of PGC-1α by SR18292.

**Methods:**

We treated BMDC cultures with ZLN005 (PGC-1α activator) or SR18292 (PGC-1α inhibitor), and LPS. We characterized the effect of PGC-1α manipulation on the metabolism of BMDCs by metabolomics and Seahorse XF Cell Mito Stress Test. In addition, we evaluated features of tolDCs, such as gene expression by qPCR, and cytokine production by ELISA.

**Results:**

We observed that activation of PGC-1α altered the metabolic profile of BMDC, and upregulates genes known to be expressed in tolDCs. However, this transcriptional upregulation was lost after LPS stimulation and did not alter TNF or IL-10 levels in BMDCs. PGC-1α inhibition, on other hand, decreased features of tolDCs, such as metabolic profile, transcriptional activation, and the levels of LPS-induced IL-10. Our data suggest that PGC-1α activation with ZLN005 does not increase the generation of tolDCs in vitro. However, inhibition of PGC-1α with SR18292 constrains tolDCs generation.

**Conclusions:**

PGC-1α is important for the development of tolDCs in murine dendritic cells.

**Funding Agencies:**

NSERC and FRQNT

